# Inhalational Toxicity of Aluminum Phosphide as an Ongoing Concern; a Report of Two Cases

**Published:** 2019-11-17

**Authors:** Azam Shafahi, Babak Mostafazadeh, Bita Dadpour

**Affiliations:** 1 Medical Toxicology Research Center, Faculty ofMedicine,Mashhad University ofMedical Sciences,Mashhad, Iran.; 2 Toxicological Research Center, Shahid Beheshti University ofMedical Sciences, Tehran, Iran.; 3 Department of ForensicMedicine and Toxicology, Shahid Beheshti University ofMedical Sciences, Tehran, Iran.

**Keywords:** Aluminum phosphide, inhalation, inhalation exposure, gas poisoning

## Abstract

Acute aluminium phosphide (ALP) poisoning is an extremely lethal poisoning. Ingestion is usually suicidal in intent, uncommonly accidental and rarely homicidal. Unfortunately, the absence of a specific antidote results in very high mortality and the key to successful treatment is in rapid decontamination and institution of resuscitative measures.

Phosphine gas is highly toxic, and fatality is expected even several hours after continuous exposure. However, intensive supportive treatments may be lifesaving in some cases. Here, two cases of accidental inhalation intoxication with ALP are reported. One patient was discharged and another suffered cardiac arrest during treatment.

## Introduction:

Aluminium Phosphide (ALP) poisoning as a suicidal attempt or accidental poisoning has become notorious in Iran over the past decades. Phosphine gas is a colorless, flammable and highly toxic substance, which is released when Phosphide comes in contact with water (moisture) or acid, with an odor of garlic or decaying fish (1-3).

By inhibition of cytochrome oxidase c and inducing oxidative stress, phosphine causes severe mitochondrial dysfunction, tissue hypoxia, and finally multiple organ failure (2, 4). 

Common clinical manifestations include gastrointestinal symptoms, progressive metabolic acidosis, refractory hypotension, and dysrhythmia (4). Most reports of ALP poisoning are following ingestion of ALP. Unfortunately, phosphine gas inhalation also may lead to life threatening toxicity, even in large areas (4-6). 

To date, no specific antidote has been defined for this rapidly fatal poisoning and supportive measures are mainly recommended. Immediate diagnosis in addition to continued intensive monitoring and supportive measures may be lifesaving. 

We aimed at reporting this incident from a public health emergency viewpoint as this type of (ALP) poisoning may reoccur (although is relatively uncommon), at least in countries where ALP is still used by general population. 

## Cases presentation:

A 60-year-old woman (case one), with her 29-year-old daughter (case two) were referred to a regional hospital and then transferred to clinical toxicology department of Imam Reza Hospital, Mashhad, Iran, with upper abdominal discomfort, nausea, and with primary suspicion to food poisoning. 

**Table1:** Laboratory findings of case one

**Laboratory parameters**	**First day**	**Second day**
Blood Sugar (mg/dl)	251	241
Creatinine (mg/dl)	1.1	1.2
Urea (mg/dl)	31	43
Sodium (mg/dl)	140	141
Potassium (mg/dl)	3.2	3.2
Calcium, Total (mg/dl)	8.9	
Alanine Aminotransferase (ALT) (units/L)	46	37
Aspartate aminotransferase(AST) (units/L)	89	61
Alkaline Phosphatase(Alp) (units/L)	239	168
Creatine phosphokinase (units/L)	124	145
Cardiac troponin	89.30	-
Magnesium (mg/dl)	2.4	-
Serum Cholinesterase	7356	-
RBC Cholinesterase	5.2	-
**Complete Blood Count **		
Red Blood Cell ×10^6^	4.56	4.20
Hemoglobin	15.1	14.1
Mean Corpuscular Volume(MCV)	98.0	91.2
Mean Corpuscular Hemoglobin(MCH)	33.1	33.6
Mean corpuscular Hemoglobin Concentration (MCHC)	33.8	36.8
White blood cell ×10^3^	7.4	6.0
Neutrophil (%)	87.4	60.6
Lymphocyte (%)	9.1	33.0
Platelets ×10^3^	183	185

**Table 2:** Venous blood gas analysis of the first patient on admission and 2, 5, and 8 hours after presenting to emergency department

**Parameters**	**On admission**	**2 hours **	**5 hours **	**8 hours **
pH	7.31	7.37	7.41	7.41
Pco2	18.2	23	25.6	36.9
Hco3	9.2	13.1	16	23.5
Base excess	-14.4	-	-	-0.1

***Case one***

Case one suffered from shortness of breath, weakness, dizziness, restlessness, and nausea on admission. Vital signs at arrival included: SBP / DBP = 100/40 mmHg, RR = 20 / minute, O2 Saturation= 93% on room air, and PR = 90 / minute.

The patient had no fever and was alert and able to answer questions. There was no history of any drug addiction. She had a history of hypertension and diabetes. QTC prolongation was the most important finding of her Electrocardiogram (ECG). Hydration, oxygen therapy, cardiac monitoring and other supportive measures were performed. Red Blood cells and Serum cholinesterase levels were in normal range and serum test for the presence of acetaminophen, ethanol, methanol, and other toxic substance were negative. Mild Metabolic acidosis was evident in the first venous blood gas (VBG). Results of other laboratory tests are shown in Table 1. Cardiology consult and echocardiography were performed and left ventricle ejection fraction (LVEF) was 35%.

With suspicion to ALP poisoning, based on clinical and para-clinical findings, magnesium sulfate, bicarbonate, N-acetyl cysteine (NAC) ​​were initiated. NAC was discontinued because of symptoms of hypersensitivity reactions. Insulin was prescribed based on the routine protocol because of high Blood Sugar. ECG was repeated and prothrombin time was also reported high, which became normal in recheck. VBG was repeated several times and improved with supportive acidosis treatment. 

***Case two***

Vital signs of daughter on arrival included: Systolic Blood Pressure (SBP)/ Diastolic Blood Pressure (DBP) = 120/80 mmHg, Respiratory Rate (R.R) = 12 / minute, Pulse Rate (P.R) = 65 / minute. The patient had a seizure attack on admission and after a short time had a cardiac arrest. She was immediately intubated and cardiopulmonary resuscitation (CPR) was performed. Unfortunately, resuscitation efforts were unsuccessful and she died after an hour. The course of events was so rapid that there was no time for any laboratory test.

***Cause of poisoning***

After taking a more accurate history it was revealed that the patient's neighbor had sprayed a pesticide in his home two days earlier and had left the house. Following investigations, it was determined that the mentioned poison was rice tablet (aluminum phosphide) which was obtained from an illegal center and was placed in the neighboring house in large numbers. The mother and daughter had been inhaling the poison odor for the past two days and had been at home all the time until they had progressive symptoms leading them to refer to hospital. History, clinical signs, and results of laboratory tests were in concordance with inhalation poisoning with aluminum phosphide. On the fourth day of hospitalization after partial recovery, case one was discharged with personal consent against medical advice.

## Discussion:

Cardiac arrest due to cardiac toxicity of ALP inhalation led to death of case two in this report. Phosphine gas toxicity following unintentional inhalation is not a common report in medical literature, although suicidal ingestion of aluminum phosphide tablets has been reported frequently in our country (2, 7, 8). 

The National Institute for Occupational Safety and Health (NIOSH) has established a limit for occupational exposure to phosphine gas at 0.3 ppm and has deemed it "immediately dangerous to life or health" at 50 ppm or more."(9). A study on different animals regarding degree of lethal exposure it has been reported that 200 ppm-hours leads to roughly 50% mortality (10). However, another study on male rats reported 11 ppm for 4 hours resulting in 50% lethality (9). 

Phosphine gas concentration in this family's home was certainly sufficient for major toxicity following several hours of exposure. An accurate estimation of fatality rates of phosphine gas inhalation is not possible. However, it is documented that phosphine is placed in the highest category of toxicity based on the Environmental Protection Agency (EPA) (9, 11, 12). 

In the current report, after 48 hours of inhalational exposure, death occurred. Unfortunately, since there is no accepted definitive treatment for this lethal poisoning, supportive measures are the only treatment option at present. Several maneuvers are suggested for possibly improving the outcome, including administration of N-acetylcysteine, magnesium sulfate, pralidoxime, and hyperinsulinemia uglycemia (13). Although none of them are sufficiently evidence advocated at the present time (6). However, intensive supportive treatments may be lifesaving in some cases, like in the current report. 

## Conclusion:

Phosphine gas is highly toxic, and fatality is expected even several hours after continuous exposure. A high level of vigilance and a more restrictive policy is needed for keeping the general population from having access to these pesticides and consequently preventing the high rate of mortality following this preventable poisoning in our community.

## References

[1] Singh I, Gupta K, Agarwal S, Bansal MK, Samad A, Kalra P (2019). Clinical efficacy of oral gabapentin versus clonidine for preemptive analgesia in knee arthroplasty under epidural anesthesia with 075% Ropivacaine–A comparative study. Indian Journal of Pain.

[2] Mehrpour O, Amouzeshi A, Dadpour B, Oghabian Z, Zamani N, Amini S (2014). Successful treatment of cardiogenic shock with an intraaortic balloon pump following aluminium phosphide poisoning. Archives of Industrial Hygiene and Toxicology.

[3] Soltaninejad K, Nelson LS, Bahreini SA, Shadnia S (2012). Fatal aluminum phosphide poisoning in Tehran-Iran from 2007 to 2010. Indian journal of medical sciences.

[4] Mehrpour O, Jafarzadeh M, Abdollahi M (2012). A systematic review of aluminium phosphide poisoning. Archives of Industrial Hygiene and Toxicology.

[5] Nosrati A, Karami M, Esmaeilnia M (2013). Aluminum phosphide poisoning: A case series in north Iran. Asia Pacific Journal of Medical Toxicology.

[6] Dadpour B, Mokhtarpour M, Abdollahi M, Afshari R (2016). An outbreak of aluminium phosphide poisoning in Mashhad, Iran. Arhiv za higijenu rada i toksikologiju.

[7] Bagheri-Moghaddam A, Abbaspour H, Tajoddini S, Mohammadzadeh V, Moinipour A, Dadpour B (2018). Using intra-aortic balloon pump for management of cardiogenic shock following aluminum phosphide poisoning; Report of 3 cases. Emergency.

[8] Dadpour B, Mehrpour O, Oghabian Z, Dabbagh Kakhki VR (2013). The feasibility of evidence-based decision making in a toxicology emergency case. Future of medical education journal.

[9] Lemoine TJ, Schoolman K, Jackman G, Vernon DD (2011). Unintentional fatal phosphine gas poisoning of a family. Pediatric emergency care.

[10] Pepelko B, Seckar J, Harp PR, Kim JH, Gray D, Anderson EL (2004). Worker exposure standard for phosphine gas. Risk Analysis: An International Journal.

[11] Proudfoot AT (2009). Aluminium and zinc phosphide poisoning. Clinical toxicology.

[12] Sudakin D (2005). Occupational exposure to aluminium phosphide and phosphine gas? A suspected case report and review of the literature. Human & experimental toxicology.

[13] Hashemi-Domeneh B, Zamani N, Hassanian-Moghaddam H, Rahimi M, Shadnia S, Erfantalab P (2016). A review of aluminium phosphide poisoning and a flowchart to treat it. Archives of Industrial Hygiene and Toxicology.

